# Green Synthesis of Copper Oxide Nanoparticles Using *Aerva javanica* Leaf Extract and Their Characterization and Investigation of *In Vitro* Antimicrobial Potential and Cytotoxic Activities

**DOI:** 10.1155/2021/5589703

**Published:** 2021-06-18

**Authors:** Fozia Amin, Fozia, Baharullah Khattak, Amal Alotaibi, Muhammad Qasim, Ijaz Ahmad, Riaz Ullah, Mohammed Bourhia, Anadil Gul, Saira Zahoor, Rizwan Ahmad

**Affiliations:** ^1^Department of Microbiology, Kohat University of Science & Technology, Kohat, Pakistan; ^2^Biochemistry Department, KMU Institute of Medical Sciences, Kohat, Pakistan; ^3^Basic Science Department, College of Medicine, Princess Nourah Bint Abdulrahman University, Riyadh 11564, Saudi Arabia; ^4^Department of Chemistry, Kohat University of Science & Technology, Kohat, Pakistan; ^5^Department of Pharmacognosy (MAPPRC), College of Pharmacy, King Saud University, Riyadh, Saudi Arabia; ^6^Laboratory of Chemistry-Biochemistry, Environment, Nutrition, and Health, Faculty of Medicine and Pharmacy, Hassan II University, B.P. 5696, Casablanca, Morocco; ^7^Beijing Key Laboratory for Green Catalysis and Separation, Department of Chemistry and Chemical Engineering, Beijing University of Technology, Beijing 100124, China; ^8^Department of Pharmaceutics, College of Clinical Pharmacy, Imam Abdulrehman Bin Faisal University, P.O. Box 1982, Dammam 31441, Saudi Arabia; ^9^Department of Natural Products and Alternative Medicines, College of Clinical Pharmacy, Imam Abdulrahman Bin Faisal University, Dammam, Saudi Arabia

## Abstract

The development of green technology is creating great interest for researchers towards low-cost and environmentally friendly methods for the synthesis of nanoparticles. Copper oxide nanoparticles (CuO-NPs) attracted many researchers due to their electric, catalytic, optical, textile, photonic, monofluid, and pharmacological activities that depend on the shape and size of the nanoparticles. This investigation aims copper oxide nanoparticles synthesis using *Aerva javanica* plant leaf extract. Characterization of copper oxide nanoparticles synthesized by green route was performed by three different techniques: X-Ray Diffraction (XRD), Fourier Transform Infrared (FTIR) Spectroscopy, and Scanning Electron Microscopy (SEM). X-ray diffraction (XRD) reveals the crystalline morphology of CuO-NPs and the average crystal size obtained is 15 nm. SEM images showed the spherical nature of the particles and size is lying in the 15–23 nm range. FTIR analysis confirms the functional groups of active components present in the extract which are responsible for reducing and capping agents for the synthesis of CuO-NPs. The synthesized CuO-NPs were studied for their antimicrobial potential against different bacterial as well as fungal pathogens. The results indicated that CuO-NPs show maximum antimicrobial activities against all the selected bacterial and fungal pathogens. Antimicrobial activities of copper oxide nanoparticles were compared with standard drugs Norfloxacin and amphotericin B antibiotics. Minimum inhibitory concentration (MIC) and minimum bactericidal concentration (MBC) of copper oxide nanoparticles were 128 *μ*g/mL against all selected bacterial pathogens. MIC of fungus and minimum fungicidal concentration (MFC) of CuO-NPs were 160 *μ*g/mL. Thus, CuO-NPs can be utilized as a broad-spectrum antimicrobial agent. The cytotoxic activity of the synthesized CuO-NPs suggested that toxicity was negligible at concentrations below 60 *μ*g/mL.

## 1. Introduction

Nanotechnology is one of the essential areas of research in modern sciences. The field of nanotechnology is expanding very rapidly, creating an incredible impact on human life including pharmaceutical, food, health, chemical industry, electronics, energy science, cosmetics, space industries, and environmental sciences [1]. The interest in nanotechnology-derived products is rapidly increasing. Nanotechnology that is the inventive innovation in the present situation can enhance human well-being and also create a great effect on the improvement of human health [2].

The synthesis process of nanomaterials and the investigation of their applications and properties are one of the inspiring parts of the advanced scientific research. Recently, science and technology have made many advances; particularly, nanotechnology helps improve an advanced concept of synthesizing nanosized material of desired size and shape [3]. Different physical and chemical methods are used for the synthesis of metal oxide nanoparticles; however, the conventionally used methods such as sol-gel, chemical reduction, and hydrothermal are costly methods and nonecofriendly by producing toxic chemicals as end products [4]. Thus, ecofriendly methods for the synthesis of metal oxide nanoparticles attracted the attention such as the use of plant extract, microorganisms, and algae. But among biological methods, the use of plant extract was more favored due to its easy handling, easy availability, cost-effectiveness, and compatibility with the biomedical application such as drug delivery, cancer treatment, antibacterial and antifungal agent, and insecticide treatment [4].

In the phytosynthesis approach, the synthesis of nanoparticles is carried out by using plant extracts as a capping and reducing agent. Among metal oxide NPs like Ni, Cu, Zn, Au, and Fe, the synthesis of CuO-NPs is considered promising NPs; they are cheaper than other Nobel metals. Metal oxide NPs like CuO have been pulled into consideration generally due to their biocidal properties and they might be utilized as an effective part of numerous biomedical applications such as biomedical imaging, drug delivery, cellular delivery, and disease treatment [5]. CuO-NPs are utilized as heterogeneous catalysts, used in drug delivery, antioxidants, anticancer, and treatment agents in the pharmaceutical field [6]. CuO-NPs are exploited in the healing of wounds and socks to give them biocidal properties. Furthermore, CuO-NPs create a great potential in industrial use including gas sensors, catalytic processes, and solar cells high-temperature superconductors [6]. Copper nanoparticles can oxidize easily to form copper oxide [7]. Copper oxide NPs are highly valuable antimicrobial particles because they possess very unusual crystal structures and possess high surface areas [8]. These nanoparticles are robust and very stable, and the shelf life of Copper oxide NPs is too long compared to other organic antimicrobial agents [8]. Various synthesis methods are used for CuO synthesis such as chemical precipitation, electrochemical reduction, microwave irradiation, and thermal decomposition plants, which attracted many researchers in green route for the synthesis of copper, but in the chemical method, the use of toxic chemicals limited its applications. Thus, the synthesis of CuO-NPs using the biological method gains more attention due to its easy availability, easy handling, elimination of cell culture, and being ecofriendly [7]. Thus, using plant extract for the synthesis of oxide NPs is already reported, *Carica papaya* [9], *Aloe barbadensis* [10], *Malva sylvestris* [11], and *Gloriosa superba* [12].

Nanoparticles (NPs) are progressively utilized to target bacteria and fungi as a contrasting option to antibiotics. Nanotechnology might be worthwhile in the treatment of microbial infections. Bacterial infections are the endless cause of chronic infections and mortality. Antibiotics played a very effective role in the treatment strategy for different infections, which are caused by bacteria due to their cost-effectiveness and intense results. Several investigations have given direct confirmation that the broad spectrum utilization of antibiotics creates generation of the strains of multidrug-resistant bacteria and fungi. The super bacteria and fungi are types of microbial strains that show resistance against all the antibiotics, which have been developed recently because of the abuse of antibiotics [13]. The main groups of antibiotics are presently being used active in three different modes on the synthesis of cell wall, translational and on the DNA replication mechanisms Bacteria are resistant to all these modes of action. The mechanisms of resistance include enzyme expression that modifies or degrade many antibiotics, such as aminoglycosides and *β*-lactamases modification of cell components, including the vancomycin cell wall resistance bacteria and ribosome that are present in bacteria resistant to tetracyclines and efflux pumps expression, which provide resistance against many antibiotics. Many resistance mechanisms produced against antibiotics are unessential for NPs because the action of NPs is directly in contact with the cell wall of bacteria and not penetrating the cell; this gives the expectations that NPs would be very less prone to promoting bacterial resistance when contrasted to antibiotics. Thus, consideration has been dropped on new and effective NP-based materials with antibacterial activity [6].

In the present study, copper oxide nanoparticles were synthesized through a green route using *Aerva javanica* plant leaf extract. The plant *Aerva javanica* (Amaranthaceae) is a perpetual herb and a tall and woolly under shrub found plentifully in all rainy seasons and generally conveyed in different areas all over the world. This plant is local to Africa and also exists in a portion of the Asian nations [14]. In conventional herb, it is utilized for many purposes such as for diabetic patients and also utilized to clear swelling [15]. The powdered form of this plant is used for ulcer. The plant seeds are utilized to alleviate migraine and furthermore stiffness. *A. javanica* is examined to possess hypoglycemic, antioxidant, antimalarial, antihlmintic, analgesic, antivenin activities and medicinal applications against kidney troubles and rheumatism [16]. *A. javanica* also showed different activities including antiviral [17], antiplasmodial [18], and antidiabetic [19]. Some important phytochemicals such as steroids [20], triterpenoids [21], sugars [22], and flavonoids [23] have been accounted for *Aerva javanica* earlier. The phytochemical flavonoids mainly act as a reducing and stabilizing agent and are responsible for the synthesis of the CuO-NPs.

To the best of our knowledge, no report is accessible on the green synthesis of CuO-NPs using leaf extract of *Aerva javanica*. The present study explores the green synthesis of CuO-NPs, their characterization, and investigation of in vitro antimicrobial and cytotoxic activities.

## 2. Materials and Methods

### 2.1. Materials

The copper (II) sulfate pentahydrate (CuSO_4_·5H_2_O) percent purity is 98%. Pure bacterial and fungal pathogen cultures were obtained from the Microbiology Department, KUST. Dimethyl sulfoxide (DMSO), Mueller-Hinton agar (MHA), potato dextrose agar (PDA), neuroblastoma (Neuro2A) cells, streptomycin, penicillin, Dulbecco media (1 g/l glucose; 2 mM glutamine), and 3-(4,5-dimethylthiazol-2-yl)-2,5 diphenyltetrazolium bromide (MTT) were used.

### 2.2. Plant Collection and Extraction

The leaves of *A. Javanica* were obtained from hilly areas of District Karak, Pakistan. The fresh leaves of *A. Javanica* were then washed in tap water, cut into tiny small pieces, and dried at room temperature under the shade. About 250 g of chopped leaves was weighed and powdered mechanically using grinder and dipped in methanol separately for 14 days. The filtrates were subjected to extraction under low pressure at 40°C by using a rotary flash evaporator to give the final extract (15.50 g). A very small amount of the extract (0.1 g/ml) is used for synthesizing CuO-NPs [24].

### 2.3. Green Synthesis of Copper Oxide Nanoparticles

For the synthesis of CuO-NPs, the optimized concentration ratio of 50 ml of aqueous 4 mM copper chloride dihydrate (CuCl_2_·2H_2_O) and 2 ml aqueous leaf extract of *A. javanica* was treated and magnetically stirred; slowly and gradually, the mixture changes its light blue color to light green color. The mixture is then subjected to heating at 80°C for 2 hours. According to reported literature, the particle size of nanoparticles increases slightly up to the range of 70–80°C and the increase becomes sharped within the range of 80–90°C; thus, we selected 80°C for 2 h on the basis of all these reasons. After drop-by-drop addition of 1 M sodium hydroxide to the mixture, sodium hydroxide comes in contact with the copper ions; the mixture changes the color spontaneously from green to brownish-black precipitate and gives an indication of the formation of CuO-NPs [24]. The brown precipitate solution was then centrifuged for 15 minutes at 10,000 rpm and repeatedly washed with deionized water after that followed by washing with ethanol to remove all the impurities that are present; then a brownish-black powder was obtained after overnight drying at 60°C in a furnace [25].

### 2.4. Characterization of Copper Oxide Nanoparticles

The synthesized CuO-NPs were characterized by using spectral-analytical techniques including X-ray Diffraction (XRD) Analysis, Scanning Electron Microscopy (SEM) Analysis, and Fourier Transform Infrared (FTIR) Microscopy.

#### 2.4.1. X-Ray Diffraction Analysis (XRD)

The synthesis of CuO-NPs was confirmed by X-ray diffractometer using CuKa as a radiation source with a wavelength of 1.5406 Å. The XRD technique was performed in the 2*θ* range of 30–70, to examine the crystalline structure and phase of copper oxide.

#### 2.4.2. Scanning Electron Microscopy Analysis (SEM) Analysis

The structural morphology, shape, and size of the synthesized copper oxide NPs was were analyzed by using scanning electron microscopy (JEOL Japan).

#### 2.4.3. Fourier Transform Infra Red Microscopy (FTIR)

This technique is used to find out the functional groups present in synthesized copper oxide NPs, because each chemical bond has an energy absorption band used to examine the structural and bond information of complex to study bonding type and their strength. The FTIR spectra of synthesized samples were obtained by using the KBr pellet method, in the range of 4000–400 cm^−1^ with a resolution of 4 cm^−1^.

#### 2.4.4. UV-Visible Spectroscopy

The initial synthesis of nanoparticles was confirmed by a UV-visible spectrophotometer (Shimadzu UV-2600) in the range of 200–800 nm.

### 2.5. Biological Assays

Stock solutions of CuO-NPs were made in DMSO 1 mg/mL concentration and then diluted to 50 *μ*g/mL, 100 *μ*g/mL, and 200 *μ*g/mL, respectively. Pure bacterial and fungal pathogen cultures were obtained from the Microbiology Department, KUST, and identified by culture and microscopic analysis. Before the susceptibility testing, the bacteria were first cultured on the surface of nutrient agar. Their fresh culture obtained was subcultured on Muller Hinton agar (MHA) for bacterial susceptibility testing of Copper oxide NPs.

#### 2.5.1. Antibacterial Activity

Fresh cultures of bacteria (adjusted to the standard 0.5 McFarland turbidity) were swabbed on the surface of MHA media using cotton swabs after solidification of media. In the agar well diffusion method, in all Petri plates, three wells were formed with the help of sterile cork borer (6 mm wells). For the antibacterial activity test at a concentration of 50 *μ*g/mL, three sets of Petri plates were prepared for all bacteria. All the plate wells have copper oxide nanoparticles, *A. javanica* extract, and dimethyl sulfoxide (DMSO) as a negative control. In the first set of plates, the well concentration of the sample was 50 *μ*g/ml, in the second set of plates, wells contain a concentration of sample 100 *μ*g/ml, and the third set of plates contain a concentration of 200 *μ*g/ml. Each plate well contained copper oxide nanoparticle, *A. javanica* extract, and DMSO as a negative control. The quantity of sample used was 50 *μ*l approximately. All the Petri plates were then placed in incubation for 24 hours at 37°C. Then zones of inhibition were measured [26].

#### 2.5.2. Antifungal Activity

The fungus was cultured on the surface of potato dextrose agar (PDA) Petri plates before being incubated at 37°C for 2-3 days. The fungus was spread on the surface of Muller Hinton agar (MHA) media using cotton swabs when solidified. For the agar well diffusion method, with the help of sterile cork borer, three wells were formed in all Petri plates. For negative control, DMSO was used. Then all the plates were subjected to incubation at 27°C for 48–72 hours; then inhibition zones were measured.

#### 2.5.3. Minimum Inhibitory Concentrations (MICs)

MIC is the lowest concentration of antimicrobial agents which inhibit microbial growth. The antimicrobial activity of copper oxide NPs was calculated using the standard broth dilution method (CLSI M07-A8). BHI broth was used for MIC calculation by twofold serial dilutions of CuO-NPs. Different concentrations were used starting from 8 *μ*g/mL to 156 *μ*g/mL with an adjusted bacterial concentration of 0.10 at 625 nm (1 × 108 CFU/ml, 0.5 McFarland's standard). Tested bacterial concentration in BHI broth was used as positive control and the only broth was used as a negative control. The incubation time and temperature were 24 h and 37°C. The MIC was calculated visually by the turbidity of the tubes before and after the incubation, and it was performed in six sets to calculate its value for the selected bacteria. After the MIC measurement of the CuO-NPs, 50 *μ*l sample from all the tubes was swabbed on plates; the plates that showed no visible bacterial growth were cultured in BHI agar plates and were incubated for 24 hours at 37°C [27].

#### 2.5.4. Minimum Bactericidal Concentrations (MBCs)

MBC is the lowest concentration of antimicrobial agent that kills all the bacteria. MBC was performed with same MIC steps. Those plates that have no bacterial growth were considered to be MBC.

### 2.6. Cytotoxic Activity of CuO-NPs

#### 2.6.1. Cell Culture

Neuroblastoma (Neuro2A) cells were incubated with 10 percent FBS, 100 *μ*g/mL streptomycin, and 100 U/ml penicillin at a temperature of 37°C and 5 percent humidity in the modified Dulbecco media (1 g/l glucose; 2 mM glutamine) [28].

#### 2.6.2. Cytotoxicity Evaluation

Using 3-(4,5-dimethylthiazol-2-yl)-2,5 diphenyltetrazolium bromide (MTT), the cytotoxic activity of prepared CuO-NPs was calculated. In short, 200 *μ*L of cell suspension was applied to a 96-well (1 to 104 cell/well) tissue culture plate and incubated at 37°C for 24 hours. Then, 50 *μ*L (0–600 *μ*g/ml) of the synthesized nanoparticles was poured into each well, while the plate was incubated for 24 hours. In this project, as the control, we identified the cell suspension that held the culture medium. Then 20 *μ*l of the MTT-containing PBS buffer (5 mg/ml) was appended to each well, while the plate was incubated at 37°C for 4 hours. At the end of each well, 100 *μ*l of DMSO was applied and their optical absorbance was measured at 570 nm, which was accomplished by applying a microplate reader. Cell viability relative to control was expressed as a percentage [28].

### 2.7. Statistical Analysis

All the experiments were performed in three repeats. All the data were calculated as a mean ± standard error, using GraphPad PRISM 6 software.

## 3. Results and Discussion

### 3.1. X-Ray Diffraction Analysis

**Figure 1:**
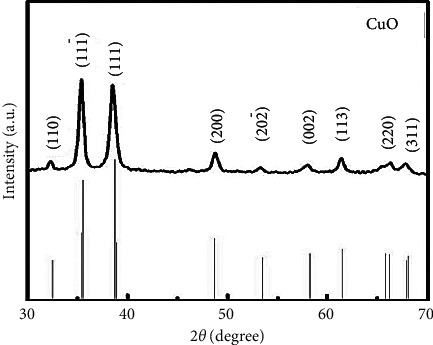
XRD patterns of copper oxide nanoparticles (CuO-NPs).

The copper oxide nanoparticles synthesized by using the green method from plant *A. javanica* are confirmed by the peaks observed (Figure 1). The peak position is at 2*θ* of 32.41, 35.61, 38.81, 48.91, 53.32, 58.22, 60.61, and 63.6, which were assigned to (110), (11¯1), (111), (200), (20^¯^2), (002), (113), (220), and (311) planes, which are in good agreement with those of CuO-NPs obtained from international center of diffraction data (ICDD) card no (801916). This confirms the formation of crystalline monoclinic morphology. Copper oxide nanoparticles show sharp and well-defined reflections on XRD patterns which give the verification of the crystalline nature of CuO nanoparticles [29].

Furthermore, the crystallite size of CuO-NPs was 15 nm, calculated by using the Debye Scherer equation (*D* *=* *kλβ*cos *θ*), where *k*, *λ*, *β*, and *θ* are Scherer constant, wavelength of X-rays (1.5418 Å), peak broadening at half the maximum intensity, and Bragg angle, respectively. Thus, the XRD analysis demonstrated that, initially, the particles are in the form of collides and then tend to grow and further react with environmental O_2_; this can be confirmed from SEM results that show some aggregates of particles; similar results were observed by Mojtaba Taran et al. [30].

### 3.2. Scanning Electron Mmicroscopy Analysis (SEM)

**Figure 2:**
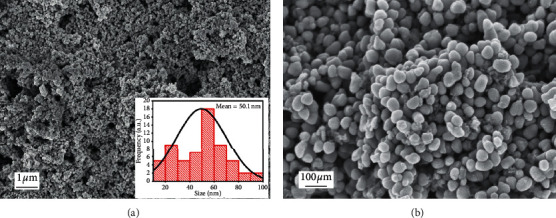
SEM micrograph of copper oxide nanoparticles.

SEM confirmed the morphology of the synthesized CuO nanoparticles.  Figure 2 shows the morphological form of CuO-NPs. From the SEM image, it is observed that the CuO nanoparticles are in highly collected form and have almost spherical morphology. The particle size of synthesized NPs was ranging from 14 to 100 nm with a mean particle size 50.1 nm, measured by using ImageJ software. The results are consistent with XRD results.

### 3.3. Fourier Transform Infra Red Microscopy (FTIR)

**Figure 3:**
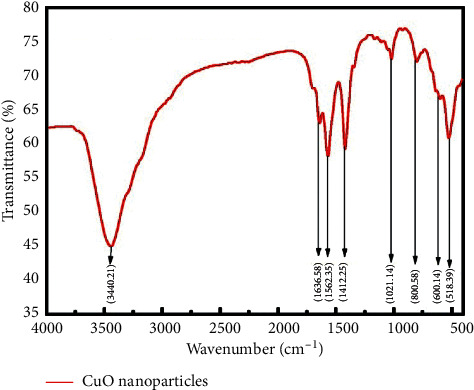
FTIR analysis of copper oxide nanoparticles.

Figure 3 showed the FTIR spectrum of the copper oxide nanoparticles in the range of 400–4000 cm^−1^ at room temperature. The Infrared (IR) spectrum of CuO-NPs shows the band at 3340.21 cm^−1^, 1636.58 cm^−1^, 1562.35 cm^−1^, 1412.25 cm^−1^, 1021.14 cm^−1^, 800.58 cm^−1^, 600.14 cm^−1^, and 518.39 cm^−1^; characteristic peaks of copper oxides are positioned between 518.4 and 1021.1 cm^−1^. The peaks observed at 518.4 cm^−1^ and 600.1 cm^−1^ indicated the formation of CuO nanostructure and Cu–O stretching. Peaks 1021.14 cm^−1^ and 800.58 cm^−1^ can be assigned to C–O and C–H bending. The peaks observed between 1412.3 and 1636.4 cm^−1^ correspond to O–H bending and C=C stretching. The peak at 3440.2 is assigned to N–H stretching which might be due to amino acid which also acts as capping agent [27].

### 3.4. Antibacterial Activity of Copper Oxide Nanoparticles

#### 3.4.1. Antibacterial Activity at a Concentration of 50 *μ*g/mL

**Figure 4:**
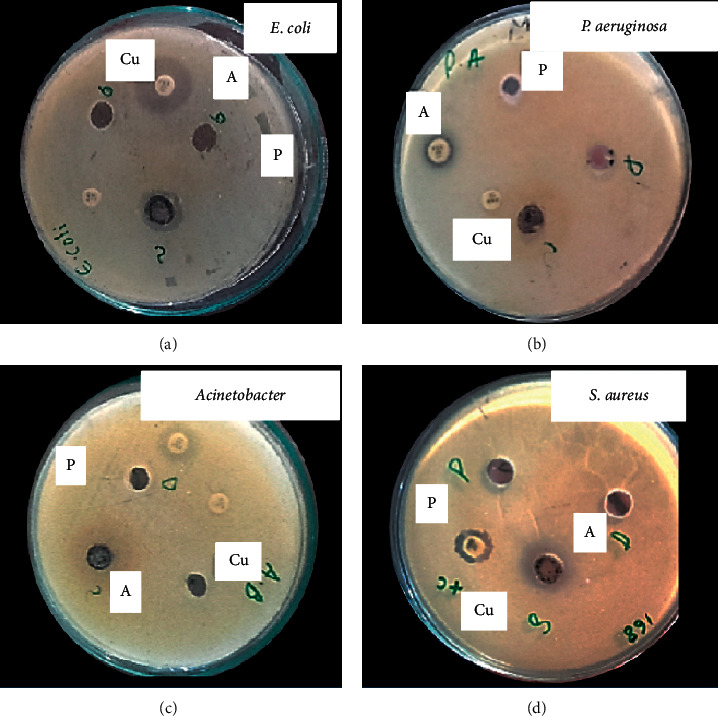
Antibacterial activity of CuO-NPs against all selected bacterial pathogens at a concentration of 50 ug/ml: (a) *E. coli*. (b) *P. aeruginosa*. (c) *A. baumannii*. (d) *S. aureus.*

Antibacterial activities of CuO-NPs were checked in vitro against *P. aeruginosa, Escherichia coli, Staphylococcus aureus,* and *A. baumannii* at a concentration of 50 *μ*g/ml (Figure 4; Table 1). DMSO was used as negative while for positive control, Norfloxacin was used. Norfloxacin is a broad-spectrum antibacterial agent. This study comprised of *in vitro* activity against a broad range of Gram-positive and Gram-negative bacterial strains; therefore, we selected this broad-spectrum drug as a positive control. It was investigated that CuO-NPs were more effective against *S. aureus*, *P. aeruginosa, A. baumannii,* and *E. coli*, respectively. The sample showed a maximum inhibition zone of 9 ± 1 mm against *S. aureus* while *A. javanica* extracts display a maximum zone of inhibition of 4 ± 1 mm against *P. aeruginosa*.

#### 3.4.2. Antibacterial Activity at a Concentration of 100 *μ*g/mL

**Figure 5:**
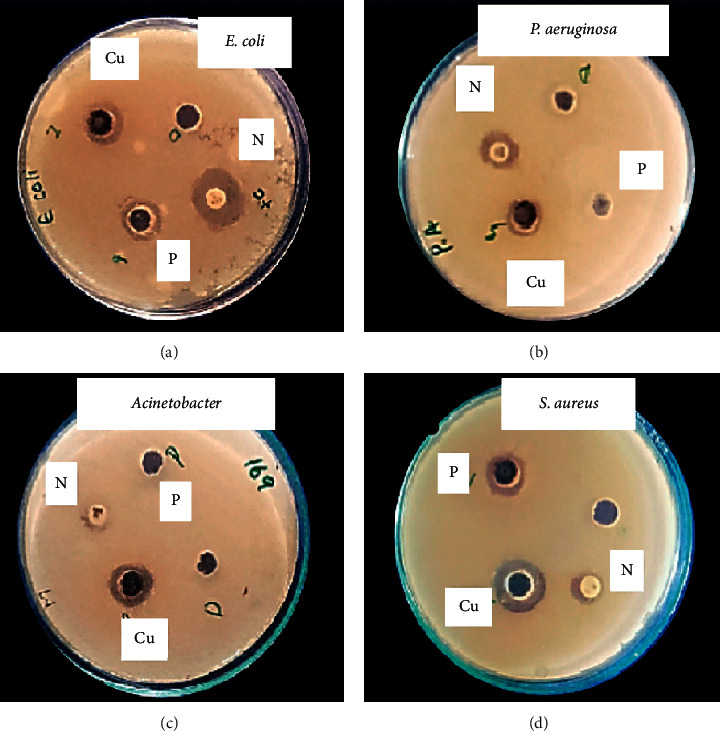
Antibacterial activity of copper oxide nanoparticle against selected bacterial strains at a concentration of 100 *μ*g/ml: (a) *E. coli.* (b) *P. aeruginosa.* (c) *Acinetobacter.* (d) *S. aureus*.

Antibacterial activities of CuO-NPs were checked *in vitro* against *P. aeruginosa, E. coli*, *S. aureus,* and *A. baumannii* at a concentration of 100 *μ*g/ml (Figure 5; Table 2). For negative control, DMSO was used and antibiotic Norfloxacin was used as a positive control. Average inhibition zones were calculated after three repeats. It was investigated that CuO-NPs are more effective against *S. aureus*, *Acinetobacter*, *P. aeruginosa,* and *E. coli*, respectively. Sample shows a maximum inhibition zone of 12 ± 1 mm against *S. aureus* while *Aerva javanica* extract showed maximum inhibition zone against all selected bacteria (4 ± 1 mm).

#### 3.4.3. Antibacterial Activity at a Concentration of 200 *μ*g/mL

**Figure 6:**
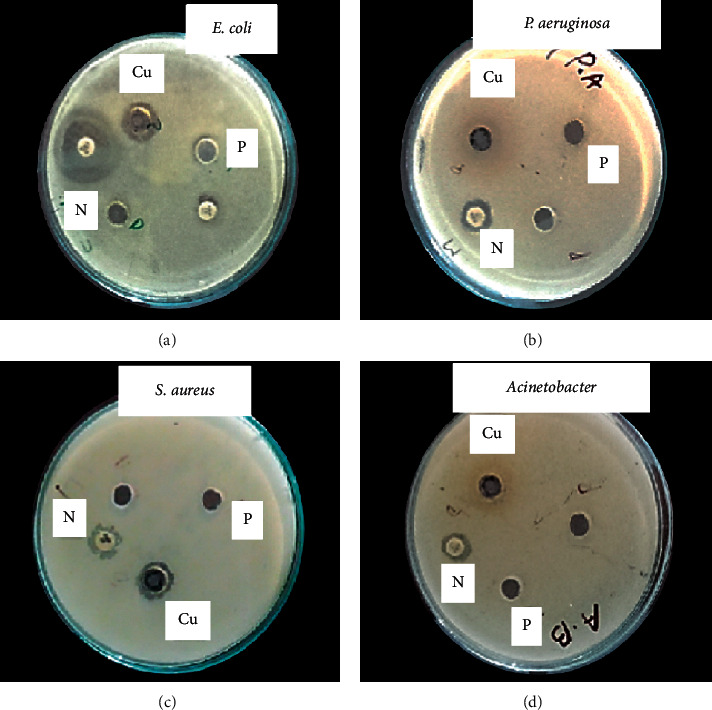
Antibacterial activity of CuO-NPs against selected bacterial strains at a concentration of 200 *μ*g/ml: (a) *E. coli.* (b) *P. aeruginosa.* (c) *S. aureus.* (d) *Acinetobacter.*

Antibacterial activities of CuO-NPs were checked in vitro against *P. aeruginosa, E. coli*, *S. aureus*, and *A. baumannii* at a concentration of 200 *μ*g/ml (Figure 6; Table 3). Antibiotic Norfloxacin was used as a positive control; DMSO was used as a negative control. It was found that CuO-NPs showed a maximum inhibition zone of 13±1 mm against *P. aeruginosa* and *Acinetobacter*.

### 3.5. Antifungal Activity of CuO-NPs

**Figure 7:**
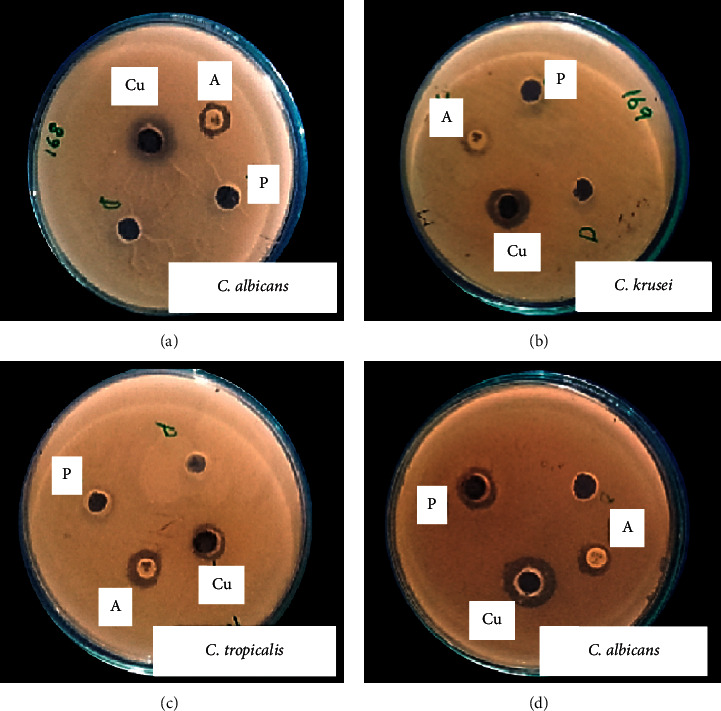
Antifungal activity of CuO-NPs against selected fungus pathogens at a concentration of 100 *μ*g/ml: (a) *C. albicans.* (b) *C. krusei.* (c) *C. tropicalis.* (d) *C. albicans*.

Antifungal activities of CuO-NPs were checked *in vitro* against *C. albicans, C. krusei,* and *C. tropicalis* at a concentration of 100 *μ*g/mL (Figure 7; Table 4). Amphotericin B antibiotic was used as a positive control and DMSO was used as a negative control. It was investigated that copper oxide nanoparticles showed a maximum zone of inhibition of 9 ± 0.5 mm against *C. albicans*.

### 3.6. Minimum Inhibitory Concentration (MIC) and Minimum Bactericidal Concentration (MBC)

#### 3.6.1. Minimum Inhibitory Concentration (MIC)

When the MIC of copper oxide nanoparticles was examined, it was concluded that it showed the highest antibacterial activity. The MIC value for CuO-NPs was at a concentration of 128 *μ*g/mL and 256 *μ*g/ml which were examined against all selected bacterial pathogens. Results confirm that the MIC was effective at a concentration of 128 *μ*g/ml (Table 5).

#### 3.6.2. Minimum Bbactericidal Cconcentration (MBC)

The copper oxide nanoparticles were effective bacteriostatic at a concentration of 128 *μ*g/mL and 256 *μ*g/mL against all the bacterial pathogens that are selected (Table 5).

#### 3.6.3. Minimum Inhibitory Concentration (MIC) for Fungus

When the MIC of CuO-NPs was performed, it was noticed that it exhibited the highest antifungal activity. The MIC value for CuO-NPs was at a concentration of 160 *μ*g/mL and 320 *μ*g/mL, which were observed against all selected fungal pathogens. Results confirm that the MIC was found to be effective at a concentration of 160 *μ*g/ml (Table 6).

#### 3.6.4. Minimum Fungicidal Concentration (MFC)

The copper oxide nanoparticle was found to be effective and fungistatic at a concentration of 160 *μ*g/mL and 320 *μ*g/mL against all selected bacteria. The result confirms the highest MFC of CuO-NPs was effective at a dilution of 160 *μ*g/ml against all the fungal pathogens that are selected (Table 6).

### 3.7. Cytotoxicity Results

CuO-NPs show incredible antibacterial properties against Gram-negative and Gram-positive bacteria. According to reported literature, in vitro various human cell lines like neuronal cells, cardiac microvascular endothelial cells, kidney cell, liver cell, and lung epithelial cells demonstrated that CuO nanoparticles cytotoxicity is mediated by oxidative stress. Thus, the excessive use of CuO-NPs and their disposal increases the risk of its potential toxicity to environmental and human health. Therefore, it necessary to determine the biocompatibility of synthesized CuO-NPs [31–34]. Hence, the cytotoxic properties of CuO-NPs were performed on Neuro2A cells using MTT assays [28]. Different doses of CuO-NPs from 0 to 600 *μ*g/ml were used to complete this examination (Table 7). Concentrations of synthesized nanoparticles below 15.62 *μ*g/mL appeared to contain lower toxicity, as observed in the findings. From this, it can be deduced that CuO-NPs are harmless and industrially applicable with low risk at concentrations below 60 *μ*g/ml [35].

## 4. Discussion

The copper oxide nanoparticles have attracted many research interests because of their significance and importance as catalyst, ceramic resistor, superconducting material, and gas sensor, as well as their roles in the pharmaceutical field and the energy sector [36]. The present study investigated the use of *A. Javanica* leaf extract for copper oxide nanoparticles synthesis. The extract of *A. Javanica* acts as a capping agent and reducing agent in the CuO nanoparticles synthesis. X-ray diffraction analysis was performed to determine the crystal structure and phase of copper oxide nanoparticles. XRD results confirmed that the particle size was 15 nm, which was in close agreement with previously published data [37, 38]. Morphology of copper oxide nano particles confirmed by scanning electron microscopy, which showed that the particles are spherical and are in 18–23 nm range. FTIR spectroscopy was carried out to identify different functional groups and biomolecules present in copper oxide nanoparticles. The result showed different peaks that indicated the formation of CuO nanostructures, Cu–O stretches, and the presence of CO_2_ in the air and hydrated CuO. In this study, the antimicrobial activity of copper oxide nanoparticle was tested against the fungal pathogen and Gram-negative and Gram-positive bacteria. The bacterial isolates, *S. aureus, P. aeruginosa*, *E. coli*, and *A. baumannii*, and fungal isolates, *Candia albicans*, were used in the study. Higher antibacterial activities were investigated against Gram-negative bacteria as compared to Gram-positive bacteria. This might be due to the thinner cell wall of Gram-negative bacteria as compared to Gram-positive bacteria; thus, the thinner wall allows easy penetration of NPs, which then causes the cell lysis [39].

According to previously investigated data, higher activities were observed against Gram-negative bacteria [40]. At the concentration of 50 *μ*g/mL, copper oxide nano particles showed the highest activity against all the selected bacteria. CuO nanoparticles showed the highest activity against *S. aureus*, while *A. baumannii*, *P. aeruginosa*, and *E. coli* showed the lowest activity. Previously investigated studies showed that *S. aureus* is less susceptible to CuO nanoparticle compared to *E. coli* [41].

At a concentration of 100 *μ*g/mL, CuO-NPs showed better activity against all the selected bacteria. Bacterial isolates *S. aureus* and *A. baumannii* showed the highest activity and *P. aeruginosa* and *E. coli* showed the lowest activity while previously published data showed that, at the concentration of 50 *μ*g/mL, *E. coli* showed the highest activity [42]. At concentration 200 *μ*g/mL, *P. aeruginosa and A. baumannii* showed the highest activity, which is in good agreement with previously published data which showed that *E. coli* and *P. aeruginosa* showed maximum activity at 250 *μ*g/ml [43]. Copper oxide nanoparticles also showed maximum antifungal activity. At a concentration of 100 *μ*g/ml, CuO nanoparticles *C. albicans* showed maximum activity. Previously published data confirm that CuO-NPs showed maximum activity against *Candida albicans* [27]. The MIC and MBC were calculated in different concentrations of copper oxide nanoparticles. Different concentrations of all samples were made. Different dilutions of CuO nanoparticles 8 *μ*g/mL, 16 *μ*g/mL, 32 *μ*g/mL, 64 *μ*g/mL, 128 *μ*g/mL, and 256 *μ*g/mL were made. At a concentration of 128 *μ*g/mL, samples inhibit the growth of all selected bacteria. So MIC and MBC for all selected bacteria were 128 *μ*g/mL [27]. MIC and MFC were calculated for fungus in different concentrations ranging from 10 *μ*g/mL to 320 *μ*g/mL. Results indicated that the MIC and MFC for all selected *Candida* species were at 160 *μ*g/Ml, which are in good agreement with previously published data [27]. Moreover, besides the concentration of synthesized nanoparticles, the antibacterial activity of nanoparticles greatly depends on their size and surface area [40, 44]. Azam et al. studied the effect of the size of CuO-NPs on their antibacterial activity and they demonstrated that the smaller nanoparticles (20 nm) have higher activity than larger size NPs. Further, the small size NPs provide greater surface area for the production of reactive O_2_ species such as hydrogen peroxide, superanion radicals, and hydroxyl radicals [42, 45].

## 5. Conclusion

It is concluded that the synthesis of copper oxide nanoparticles by a green approach using *Aerva javanica* extract is inexpensive, very easy to carry out in any laboratory, and also nontoxic. XRD analysis confirmed the crystalline structure of CuO nanoparticles and investigated the size in the range of 15 nm. SEM confirmed the spherical shape and particle size in 18 to 23 nm which is in good agreement with previously providing data. The FTIR confirmed different function group and physical interaction of macromolecules with CuO-NPs. The CuO nanoparticles showed desirable antibacterial, antifungal, and cytotoxic activities. In line with the findings of the inquiry into the toxicity of synthesized nanoparticles, these nanoparticles tend to be at low risk in terms of their use in the industry as long as they are applied at concentrations below 60 *μ*g/mL. However, further *in vivo* studies will explore their potential in using these against microbial infectious diseases.

## Figures and Tables

**Table 1:** Different inhibition zone of CuO-NPs against selected bacterial strains.

—	Inhibition of zone (mm) against fungal strains			
—	*E. coli*	*P. aeruginosa*	*S. aureus*	*Acinetobacter*
CuO-NPs	5 ± 1	5 ± 1	9 ± 1	5 ± 1
*Aerva javanica* leaf extract	2 ± 1	0	0	0
Norfloxacin	12 ± 0	5 ± 0	4 ± 0	3 ± 1
DMSO	0	0	0	0

Norfloxacin as a positive control; DMSO = dimethyl sulfoxide as negative control; ± standard error mean.

**Table 2:** Different zone of inhibition of CuO-NPs against selected bacterial strains.

—	Inhibition zone (mm) against fungal strains			
—	*E. coli*	*P. aeruginosa*	*S. aureus*	*Acinetobacter*
CuO-NPs	6 ± 1	10 ± 1	12 ± 1	12 ± 1
*A. Javanica* leaf extract	4 ± 1	3 ± 1	3 ± 1	2 ± 0.25
Norfloxacin	14 ± 1	5 ± 0	5 ± 0	5 ± 1
DMSO	0	0	0	0

Norfloxacin as a positive control; DMSO = dimethyl sulfoxide as negative control; ± standard error mean.

**Table 3:** Different inhibition zones of all samples against selected bacterial strains.

—	Inhibition zone (mm) against fungal strains			
—	*E. coli*	*P. aeruginosa*	*S. aureus*	*Acinetobacter*
CuO-NPs	7 ± 0.57	13 ± 1	12 ± 1	12 ± 1
*Aerva javanica* leaf extract	5 ± 0.35	4 ± 0	5 ± 0.35	4 ± 0
Norfloxacin	15 ± 1	5 ± 0	5 ± 1	6 ± 0
DMSO	0	0	0	0

Norfloxacin as a positive control; DMSO = dimethyl sulfoxide as negative control; ± standard error mean.

**Table 4:** Different inhibition zones of all samples fungal pathogens *Candida albicans*.

—	Inhibition zone (mm) against fungal strains			
—	*C. albicans*	*C. krusei*	*C. tropicalis*	*C. albicans*
CuO-NPs	9 + 0.5	5 + 1	4 + 0	7 + 1
*Aerva javanica* leaf extract	0	0	3 + 0	3 + 0.5
Amphotericin B	4 + 1	3 + 0	3 + 1	3 + 0
DMSO	0	0	0	0

**Table 5:** MIC and MBC determination of coppe oxide nanoparticles.

Bacterial strains	Concentrations					
	256 *μ*g/mL	128 *μ*g/mL	46 *μ*g/mL	32 *μ*g/mL	16 *μ*g/mL	8 *μ*g/mL
*Escherichia coli*	−	−	+	+	+	+
*Pseudomonas aeruginosa*	−	−	−	+	+	+
*Staphylococcus aureus*	−	−	+	+	+	+
*Acinetobacter baumannii*	−	−	−	+	+	+

Positive (+) = growth; negative (−) = no growth.

**Table 6:** MIC and MFC determination of copper oxide nanoparticles.

Fungal strains	Concentrations					
	320 *μ*g/mL	160 *μ*g/mL	80 *μ*g/mL	40 *μ*g/mL	20 *μ*g/mL	10 *μ*g/mL
*C. albicans*	−	−	+	+	+	+
*C. krusei*	−	−	+	+	+	+
*C. tropicalis*	−	−	+	+	+	+
*C. albicans*	−	−	+	+	+	+

Positive (+) = growth; negative (−) = no growth.

**Table 7:** MTT assay results of synthesized CuO-NPs at 550°C.

S. no.	Concentration *μ*g/mL	Cell visibility (%)
1	0	100
2	600	35
3	300	37
4	150	44
5	125	45
6	100	60
7	70	60
8	60	80
9	50	80
10	30	80
11	10	80

## Data Availability

All the available data are included within the article.
